# Proteomic Analysis Highlights Peculiar Protein and Phosphoprotein Profiles in Dermal Fibroblasts from Celiac Disease Patients

**DOI:** 10.3390/ijms27093938

**Published:** 2026-04-28

**Authors:** Antonio Montefusco, Maria Laura Bellone, Antonio Massimiliano Romanelli, Merlin Nanayakkara, Maria Vittoria Barone, Fabrizio Dal Piaz, Ivana Caputo, Gaetana Paolella

**Affiliations:** 1 Department of Medicine, University of Salerno, 84084 Fisciano, Italy; amontefusco@unisa.it (A.M.); mbellone@unisa.it (M.L.B.); fdalpiaz@unisa.it (F.D.P.); 2 Department of Engineering, University of Sannio, 82100 Benevento, Italy; aromanelli@unisannio.it; 3 Federico II University Hospital, 80131 Naples, Italy; merlinnanayakkara@yahoo.it; 4 European Laboratory for the Investigation of Food-Induced Diseases (ELFID), University Federico II, 80131 Naples, Italy; mv.barone@unina.it; 5 Department of Translational Medical Science, University Federico II, 80131 Naples, Italy; 6 Department of Chemistry and Biology, University of Salerno, 84084 Fisciano, Italy

**Keywords:** celiac disease, skin fibroblasts, celiac cellular phenotype, comparative proteomics, phosphoproteomics

## Abstract

Celiac disease (CD) is an autoimmune inflammatory enteropathy triggered by consuming gluten-containing cereals. A key role in its pathogenesis is played by type 2 transglutaminase, an enzyme that causes an increase in gluten immunogenicity. Celiac cells seem to present constitutive characteristics, even recognizable under a gluten-free diet, such as defects in vesicular trafficking and autophagy, protein hyperphosphorylation, and cytoskeleton rearrangement. In this work, by using an omics approach, we attempted to identify those proteins differentially expressed or differentially phosphorylated in a cell model suitable to study cell behavior in the absence of inflammation, i.e., primary cultures of dermal fibroblasts from control or CD subjects. By performing mass spectrometry analyses, we found several up- and-down expressed or phosphorylated proteins in CD samples, mainly involved in signaling, homeostatic responses, cytoskeleton organization, vesicular trafficking, and extracellular vesiculation. These proteins may represent a molecular signature of the celiac cellular phenotype and may contribute to adding new insight into the comprehension of the complex mechanisms of CD pathogenesis.

## 1. Introduction

Celiac disease (CD) is an immune-mediated gastrointestinal disease that affects about 1% of the world’s population. It is triggered by the ingestion of gluten-containing cereals in genetically predisposed individuals [1]. In CD subjects, gluten activates an innate and an adaptive immune response that ultimately leads to gastrointestinal inflammation and manifestations, such as villous atrophy, crypt hyperproliferation, lymphocyte infiltration, and intestinal altered permeability [1]. A key role for pathogenesis is played by type 2 transglutaminase (TG2), an enzyme that, by deamidating to glutamic acid some specific glutamine residues of gluten peptides, causes an increase in gluten immunogenicity. TG2 transamidation between glutamine and lysine residues belonging to self-proteins is also responsible for mounting an autoimmune response, where TG2 is the main autoantigen. Frequently, several extraintestinal manifestations affect CD patients, and there are also clinical manifestations in the absence of gluten ingestion [2], demonstrating that CD pathogenesis is not limited to the gastrointestinal compartment but is a systemic disease. Some studies have highlighted constitutive features in celiac cells far from the principal site of inflammation and/or independent from gluten exposure, leading to the concept of the existence of a “celiac cellular phenotype” [3,4,5,6]. In relation to this, skin-derived fibroblasts from CD patients on a gluten-free diet showed a higher content of phosphorylated proteins and a higher number of focal adhesions than cells from control subjects [4,7]. CD enterocytes and skin fibroblasts also showed a delay in the progression of the vesicular early endosomes to the late endosomes, associated with increased signaling, actin rearrangement, and augmented proliferation [3,7]. In addition, skin-derived CD fibroblasts appeared less adaptable to a stress condition such as the exposure to thapsigargin, a drug able to induce the activation of endoplasmic reticulum (ER) stress and the increase in autophagic markers [6]. Finally, anti-TG2 antibodies failed to protect CD skin-fibroblasts from the toxic effects of 31–43 alpha-gliadin peptide [8]. Among emerging strategies to understand the molecular mechanisms at the basis of diseases, there are those based on proteomic approaches. The quantification of proteins and characterization of their post-translational modifications relevant in disease and healthy cells contribute to a better understanding of dysregulated pathways responsible for pathogenesis. A proteomic analysis has been conducted on gut biopsies from CD subjects with active disease and in remission [9,10]. This approach led to the observation of an increased level of proteins involved in cellular metabolism, such as enzymes CYP3A4, CYP2C9, and LCT, and proteins important for microvilli formation, such as scinderin and espin, in CD subjects with active disease with respect to subjects on a gluten-free diet [10]. Proteomic analysis could also be a good tool to support CD diagnosis by defining a proteome score that can support protein–protein interaction network analysis [11]. From this approach emerged important proteins that could be involved in CD pathogenesis, such as proteins related to antigen processing and presentation (HSPA5, HSPA8), to protein folding in the ER (HSP90B1, HSPA5, HSPA8) and to legionellosis (HSPA8) [11]. In the attempt to discover novel molecular non-invasive biomarkers for CD, a recent proteomic study identified three diagnostic candidate proteins (FGL2, TXNDC5, and CHGA) differentially expressed in duodenal and plasma samples of CD patients [12]. Another proteomic analysis on plasma samples identified two proteins significantly associated with CD (CPA2 and ITGB7), which were invariant to gluten containing diet [13].

In this study, we conducted a comparative mass spectrometry (MS)-based proteomic analysis of fibroblasts derived from the skin of CD patients on a gluten-free diet and control subjects, to improve our understanding of the characteristics of the celiac cell phenotype. To this aim, the dermal model represented a unique model, precisely because skin fibroblasts are far from the intestine, where inflammation is triggered by gluten. For this reason, all differences found between control and celiac samples may contribute to the characterization of the constitutive celiac cellular phenotype, i.e., an ensemble of features that are not related to gluten exposure or to inflammation, but can contribute to explaining the molecular mechanism of susceptibility to develop the pathology. Our findings highlighted baseline differences between the control and CD groups. Indeed, they revealed significant differences in the abundance of proteins involved in critical cellular processes, including stress response, organelle structural organization, and extracellular vesicle formation, between celiac and control cells. Given the importance of protein phosphorylation in inflammatory diseases [14] and the reported evidence of altered phosphoprotein levels in intestinal cells of CD patients [15,16], we also performed phosphoproteomic analysis. Interestingly, we identified several proteins that were phosphorylated differently in CD cells compared to control cells, despite similar expression levels in both groups. To our knowledge, this is the first study investigating the proteomic profiles of cells distant from the main site of inflammation in CD. Our results confirm the presence of a distinctive CD cell phenotype and provide additional information for its characterization at a molecular level.

## 2. Results

### 2.1. Whole Proteome Analysis

**Figure 1:**
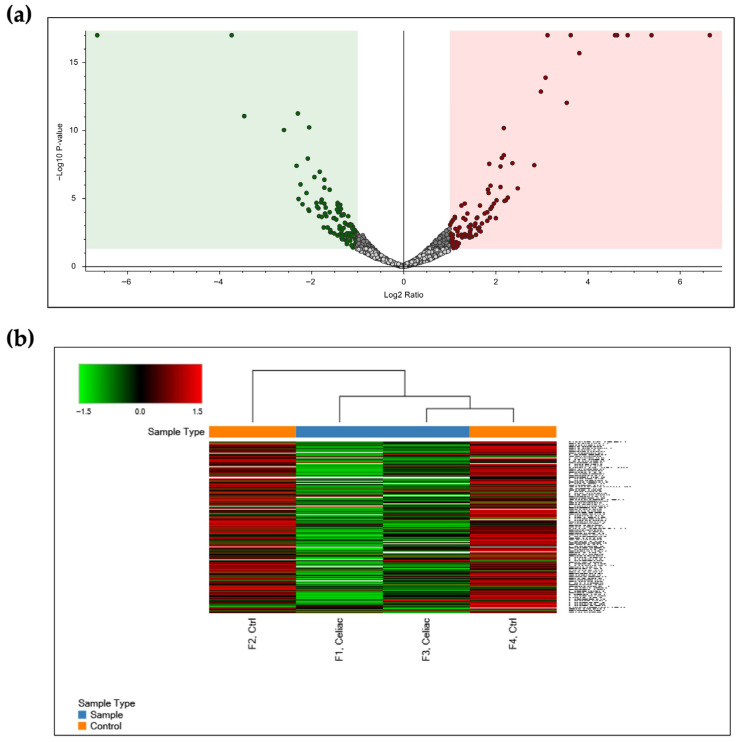
Global proteome analyses of proteins from control and CD skin fibroblasts. (**a**) Volcano plot based on significantly (*p*-value < 0.05) differentially abundant proteins collected in the green and red boxes, respectively, for abundance ratio < 0.5 and for abundance ratio > 1.8. (**b**) Heat map representation of all proteins identified in samples from control (orange) and CD patients (blue). Each lane represents the result of analysis carried out on each gel lane (two for each sample).

To identify constitutively differently expressed proteins (DEPs) in CD cells with respect to control ones, we extracted total proteins from skin-derived fibroblasts from four CD patients on a gluten-free diet and from four control subjects. Before MS analysis, samples from each group were pulled with the aim of attempting to minimize biological variation. Of the identified 3269 proteins, we evaluated their relative abundance. The resulting volcano plot (Figure 1) confirmed the reliability of the obtained data. In more detail, for 177 proteins, a significantly different abundance (*p*-value < 0.05) in the two cell types was observed, and these proteins were therefore considered DEPs. Among them, 78 proteins were present at a higher level in the CD sample (abundance ratio > 1.8) (Table S1a), and 99 proteins were more abundant in the control one (abundance ratio < 0.5) (Table S1b).

**Figure 2:**
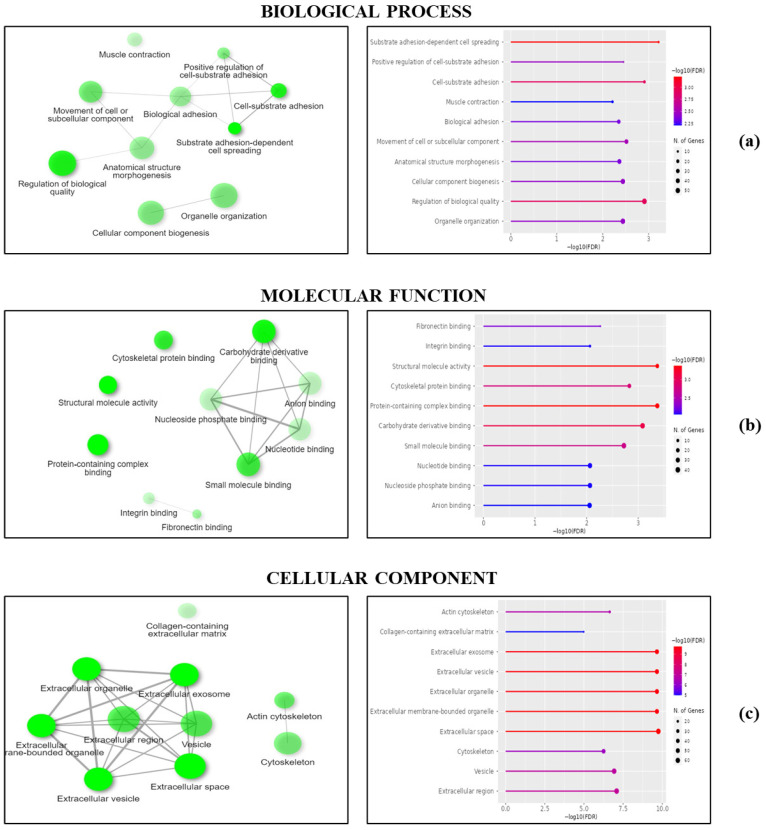
Gene Ontology analysis of DEPs. Proteins whose abundance significantly changed in CD cells compared to control ones were grouped based on the biological processes they were involved in (**a**), their molecular functions (**b**), and the cellular component where they are mainly localized (**c**).

On these identified DEPs, we performed a protein–protein interaction network analysis to visualize involved molecular pathways and functional categories, as described by Gene Ontology. In particular, proteins were associated with some specific biological processes (Table S2a), molecular functions (Table S2b), and cellular component categories (Table S2c). Among all the nodes that could be organized, we focused our interest on those including more than 20 genes. In the biological process category, most DEPs were involved in the regulation of biological quality, cellular component biogenesis, and organelle organization (Figure 2a), whereas in the molecular function category, DEPs showed structural molecule activity, carbohydrate derivative binding, and small molecule binding (Figure 2b). In the cellular component category, proteins localized in vesicles, extracellular vesicles, and organelles were listed (Figure 2c). However, almost all DEPs were responsible for intra- and intercellular communication, signal transduction, and stress response.

It is very interesting to note that when analyzing only the overexpressed or underexpressed proteins in samples from celiac patients using Gene Ontology, the results obtained are poorly informative (Figure S1). Indeed, in the first case, it was not possible to identify the biological processes, and in the second case, the molecular functions associated with these proteins. This evidence suggests that, rather than an over- or under-activation of specific pathways, the cells of patients with CD exhibit a regulation of certain processes that differs from that of fibroblasts from healthy patients.

**Figure 3:**
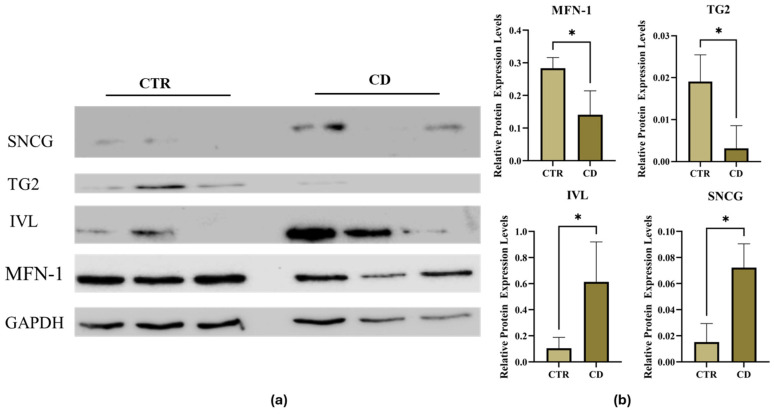
Validation of DEPs by Western blot analysis. (**a**) Representative Western blot regarding total protein levels of indicated proteins from three CD and three control (CTR) fibroblast cultures (50 μg each); GAPDH was used as the internal reference. (**b**) Densitometric analysis of protein expression; protein levels were normalized toward GAPDH expression. Data are reported as mean ± SE. * *p*-value < 0.05.

To validate MS data, we performed a Western blot analysis on single cultures. For validation, we selected mitofusin 1 (MFN1) and TG2, among proteins with reduced expression in CD samples with respect to controls. We also chose involucrin (IVL) and γ-synuclein (SNCG), among proteins with increased expression in CD samples with respect to controls. On the whole, Western blot findings confirmed MS data, even if individual variability in the expression level was observed in each group (Figure 3).

### 2.2. Phosphoproteome Analysis

**Figure 4:**
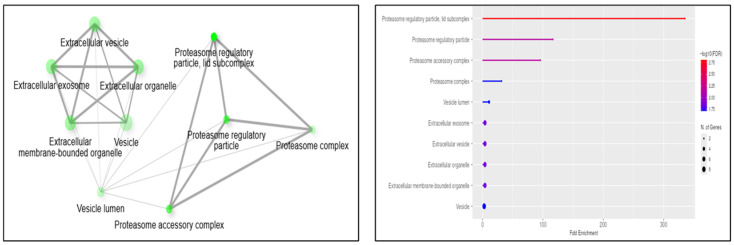
Gene Ontology analysis of differential phosphorylated proteins. Proteins whose phosphorylation level significantly changed in CD cells compared to control ones were grouped based on the cellular component where they are mainly localized.

The analysis of the whole proteome highlighted that several proteins involved in signaling were differently expressed. However, to obtain further information on the actual activation (or repression) of specific cellular pathways, we also performed a phosphoproteomic analysis. This approach was aimed at identifying those proteins whose phosphorylation state or level was significantly different in CD cells compared to the non-pathologic ones. In the attempt to reduce the interindividual variations and to make the most frequent modification more evident, in this analysis the protein samples were obtained from a pool of healthy controls or of CD patients. Protein samples were first enriched in phosphoproteins by affinity purification, fractionated by SDS-PAGE (Figure S2), digested, and analyzed by MS. This approach allowed us to identify 832 phosphoproteins. Among those proteins, the ones with more than two unique peptides and presenting a significantly different abundance (*p*-value < 0.05) in the two cell types, 14 were more abundant (abundance ratio > 2) (Table S3a), and 12 were less abundant (abundance ratio < 0.5) (Table S3b) in CD samples with respect to controls. Of these 26 differentially phosphorylated proteins, 14 also emerged from whole proteomic analysis (highlighted in Table S3a,b); among them, all proteins, except two (accession n. B4DIT7 and P35270), did not significantly vary in their expression level, indicating that the differences we found in the phosphorylation level were not due to different protein expression. Noteworthy is that most of them can be associated with vesicular and extracellular vesicular components and the ubiquitin-proteasome system, as emerged from the Gene Ontology analysis (Figure 4).

Our analysis demonstrated that one of the down-phosphorylated proteins identified by MS analysis, which was also less expressed in CD cells, was related to the cDNA FLJ58187, described as highly similar to protein-glutamine gamma-glutamyltransferase 2 (i.e., TG2) in the UniProt database. An alignment was performed between the amino acid sequence derived from this transcript and the TG2 sequence using its UniProt accession code P21980. Except for a missing segment, the alignment revealed similarities between the two sequences. Furthermore, the three-dimensional structure of this sequence was predicted using Phyre2 software, and the structure appears to be highly correlated with that of TG2 (Figure S3). Besides TG2, only another phosphorylated protein appeared differently expressed in CD and control cells, the sepiapterin reductase, an enzyme involved in the biosynthesis of tetrahydrobiopterin [17]. The upregulation of this enzyme and its differential phosphorylation could be implicated in a kind of depigmentation of the skin, vitiligo, frequently associated with CD manifestations [17,18].

## 3. Discussion

CD is a pleiotropic disease with different intestinal and non-intestinal manifestations. A gluten-free diet, in most cases, leads to remission from the disease; however, the presence of some clinical manifestations, even in patients on a gluten-free diet, suggests that celiac cells have constitutive differences that may be independent of gluten ingestion. During the years, several studies have been published describing peculiar basal features of CD cells and tissues (from individuals on a gluten-free diet) with respect to controls, and also a differential response to stressor agent, gluten, or anti-TG2 antibodies [3,4,5,6,7]. In this context, skin-derived cells from CD subjects represent an interesting and suitable in vitro model to highlight basal alteration in molecular processes, independent of gluten exposition and in the absence of inflammation. This type of model could also be useful for studies regarding the role of fibroblasts in dermatitis herpetiformis, the cutaneous manifestation of CD.

Proteomic screening could be a rapid method to identify proteins crucial in constitutively altered pathways. For this reason, in the present study, we used a comparative proteomic approach to identify DEPs in CD and control skin-derived fibroblasts. Our methodological approach included the preparation of pools from CD or control primary cultures before MS analysis, in an attempt to reduce the biological variability of the results. Even if the pooling strategy determined the loss of information about inter-individual variability and a global reduced sensitivity, it represented the more adequate option to perform analysis on a limited number of samples.

We found 78 proteins more expressed and 99 proteins less expressed in CD cells than in control fibroblasts. The robustness of our MS analysis was confirmed by a validation performed with a different technique, consisting of a Western blot analysis with antibodies recognizing specific proteins identified by MS. Western blot methodological validation indicated that the proteomic analyses gave realistic results, even if, as expected, a certain degree of variability was present in each group.

Some proteins or family members that we found increased in CD skin fibroblasts as those from the following genes, HLA-A, DOCK5, CLIC4, ALDH1A3, MAP4K4, were also upregulated in the small intestinal biopsies from CD patients (HLA-A, DOCK2, CLIC5, ALDH1L2 and ALDH4A1, MAPK10 and MAPK8) [10], indicating that skin-derived fibroblasts in part displayed the typical phenotype of celiac cells in the active phase of the disease and present in the main site of inflammation.

Interestingly, TG2, a key enzyme in CD pathogenesis, appeared less expressed in celiac skin fibroblasts than in control samples (abundance ratio of 0.2). This result could depend on both a reduced expression of the protein, as well as on its accelerated degradation/secretion. A slight, even if not significant, reduction has been previously reported in the same cell model [8], where peculiar features regarding TG2 were also observed, such as a preferential association to the cell membrane surface and to early endosomal and autophagic compartments, and a different pattern of enzymatic activation, which could significantly affect the proper autophagic flux in CD cells [5,6]. Our analysis also showed the presence of phosphorylated TG2 both in control and in CD cells, with a reduction in phosphorylation level probably due to a lower expression level in CD fibroblasts. Since the modulation of TG2 phosphorylation has been reported to have a role in mediating NFkB activation and regulating cancer growth [19] or regulating autophagy in renal cells [20], it is possible that TG2 phosphorylation could also be a mechanism of regulation of cell responses in CD cells when inflammation occurs. Altogether, our data support the concept that TG2 has a key role in determining the celiac cellular phenotype.

Several DEPs, emerging from our proteomic and phosphoproteomic analysis, were associated with vesicular compartments and were important to regulate the trafficking of signaling proteins. A lot of them belonged to extracellular compartments, such as exosomes, or to other types of vesicles, and are all involved in intercellular communication to maintain cell homeostasis. Interestingly, it has been proposed that the number and the composition of circulating extracellular vesicles could represent novel biomarkers for active CD [21]. Among upregulated DPCs in CD cells, we found glypican-1 (GPC1), a GPI-anchored protein belonging to the exosome compartment, where it interacts with several signaling molecules [22]. This protein has also been found specifically enriched on exosomes from cancer cells [23]. Other DEPs belonged to the endocytic compartment. For example, the downregulated MON2 homolog protein (MON2) modulates the transport to the Golgi through recycling endosomes [24]. The Golgi-associated PDZ and coiled-coil motif-containing protein (GOPC) has also been found localized at the trans-Golgi network, where it binds to a variety of receptors, participating in their trafficking [25]. In the case of the charged multivesicular body protein 7 (CHMP7), implied in the sorting of cargo proteins to intraluminal vesicles of multivesicular bodies, it has been reported that its upregulation may disturb the endosome–lysosome degradation pathway [26]. It is worth noting that a hyperphosphorylated protein we found in CD cells is the γ-soluble NSF attachment protein, a key protein in the disassembly of SNARE complexes in endosomal pathways [27]; phosphorylation could negatively affect NSF function, thus contributing to defective vesicular trafficking [28]. Overall, the alteration in proteins involved in vesicular trafficking, together with the observation that endosomal and autophagic trafficking were delayed in skin-derived fibroblasts [3,6], indicated the importance of vesicular compartments to define the CD-specific cellular phenotype.

A wide and important group of proteins, here identified as DEPs, is involved in the control of biological quality. Among them, proteins related to cytoskeleton regulation, protein folding, and Ca^2+^ homeostasis can be included. In this regard, a constitutive feature described in celiac skin fibroblasts was an altered Ca^2+^ homeostasis; indeed, these cells displayed lower levels of Ca^2+^ in cytosol and ER with respect to control cells [6]. An example of alteration of regulators of cytosolic Ca^2+^ concentration is the pro-chloride intracellular channel protein 4 (CLIC 4) [29], found here upregulated in CD cells. Protein chaperons also emerged as DEPs from our analyses, for example, the upregulated heat shock protein beta-7, the down-regulated isoform 2 of T-complex protein 1 subunit theta (CCT8), and the hyperphosphorylated prefoldin subunit 3. A constitutive alteration of the unfolded protein response has also been described in CD skin fibroblasts [6], and an upregulation of markers of the unfolded protein response has been recently demonstrated in CD intestinal biopsies [30]. Finally, protein degradation could be differently regulated in CD and control cells, as suggested by our findings on the different level of phosphorylation of some proteins related to the ubiquitin-proteasome function. Thus, proteomic data on alterations in proteins controlling Ca^2+^ homeostasis, protein folding, and quality control reinforce the idea that these processes could be relevant for the celiac phenotype.

Regarding cytoskeleton organization, another relevant group of DEPs emerging from our analysis was implicated in actin remodeling. Structural cellular alterations regarding cell shape and actin rearrangement have already been described as constitutive elements of the celiac cellular phenotype in fibroblasts and circulating immune cells [7,31]. In the present study, we found an increased level in CD cells of transgelin-3 (TAGNL3), an actin-binding protein that can alter the structure and the morphology of the cytoskeleton [32]. The myosin light chain kinase (MYLK) was also upregulated in celiac fibroblasts; this kinase may be implicated in many inflammatory diseases and in cancer [33]. Desmin upregulation in skin fibroblasts could be associated with inflammatory stimuli and could be related to a switch to a myofibroblast phenotype [34]. It is worth noting that the presence of an autoimmune response to desmin has been reported in CD [35]. Finally, we found in CD fibroblasts a lower phosphorylation level of utrophin, a protein that stabilizes the actin cytoskeleton and whose function can be modulated by phosphorylation [36].

Previous findings also indicated that CD skin fibroblasts displayed a defective autophagy [6]. From proteomic analysis it emerged that proteins involved in the autophagic pathway were present in both increased and decreased DEPs. Among overexpressed proteins, we found γ-synuclein (SNCG), which might play an important role in autophagy by protecting cells from ER stress [37]. A downregulated protein in CD cells was the above-cited GOPC, a protein also displaying a scaffolding function that stabilizes the VPS34/BECN1 complex during autophagy induction [38]. The glycylpeptide N-tetradecanoyltransferase 1 (NMT1), necessary for lysosomal degradation and mTORC1 activation [39], appeared downregulated in CD cells. We also found a lower expression level in CD fibroblasts of mitofusin-1 (MFN1), a protein involved in mitochondrial fusion and mitophagy activation [40]; this last one is an important process that allows the release of proinflammatory cytokines such as IL-1α, IL-1β, and IL-18 [41]. IL-18 is present in the initial steps of CD pathogenesis and is induced after in vitro gluten stimulation of intestinal biopsy from gluten-free-diet patients [42]. In our proteomic analysis, IL-18 was found to be a constitutively increased DEP. Together with MFN1 downregulation, the increase in IL-18 suggested that CD skin fibroblasts could have an altered regulation of the inflammatory process. The increase in involucrin levels we found in CD fibroblasts could also be indicative of an increased propensity to inflammation [43].

In conclusion, proteomic analyses employed in this study permitted the identification of altered protein expression and phosphorylation, which were contributing to the definition of the complex celiac cellular phenotype, consisting of a set of constitutive characteristics concerning all the cells of a subject, even if not related to the primary site of inflammation. To this regard, the dermal fibroblast model represents a singular model to point out peculiar traits unrelated to gluten exposure. Even if the data presented here are mainly descriptive and based on a limited number of primary dermal cultures, our omics approach brought out several DEPs implied in previously described phenomena related to the CD phenotype, such as Ca^2+^ homeostasis, stress response, signaling, autophagy, cytoskeleton rearrangement, and vesicular trafficking and extracellular vesiculation, which were already described in celiac skin fibroblasts and other body samples [3,4,5,6,7]. Overall, this study may contribute to adding new insight into the comprehension of the complex mechanisms of CD pathogenesis. Moreover, the identified DEPs may represent a molecular signature of the celiac cellular phenotype also in the absence of inflammation and of the immune response to dietary gluten.

## 4. Materials and Methods

### 4.1. Cell Cultures

Fibroblasts were obtained from skin biopsies of four CD patients on a gluten-free diet (age range 17–43) and four HLA-DQ2/8-negative healthy subjects (age range 25–30 years). CD patients were on a gluten-free diet for at least 4 years and showed normal (Marsh T0) biopsy, negative serology, and negativity for anti-endomysium antibodies. None of the CD patients were affected by dermatitis herpetiformis. Cells were cultured in Dulbecco’s Modified Eagle’s Medium supplemented with 20% (*v*/*v*) fetal bovine serum, 1 mM L-glutamine, 50 U/mL penicillin, and 50 µg/mL streptomycin (Invitrogen SRL, Milan, Italy). Cells were maintained at 37 °C in a 5% CO_2_, 95% air-humidified atmosphere. Fibroblasts were employed at passages between 4th and 7th.

### 4.2. Protein Samples Preparation

Protein extracts were prepared from each culture. Specifically, 1 × 10^6^ cells were mechanically harvested in lysis buffer containing 50 mM Tris-HCl, pH 7.5, 1% Triton-X 100, 2 mM EDTA, pH 8.8, 150 mM NaCl, and a protease inhibitor cocktail (Thermo Fisher Scientific, Monza, Italy). Samples were kept on ice for 60 min with gentle shaking, then centrifuged at 16.000× *g* for 20 min at 4 °C. Supernatants were collected and stored at −80° C until further analysis. For phosphoproteomic analysis, phosphoproteins were first enriched by using an affinity purification step according to the manufacturer’s protocol (PierceTM phosphoprotein enrichment kit, Thermo Fisher Scientific). After enrichment, phosphoproteins were resolved on a 12% SDS-PAGE, fixed with 50% methanol and 10% acetic acid solution for 30 min, and stained with the Pro-Q™ Diamond Phosphoprotein Gel Stain (Thermo Fisher Scientific). Finally, gel was destained in a solution of 20% acetonitrile and 50 mM sodium acetate, pH 4, and images of stained phosphoproteins were acquired by using the iBright image system (Thermo Fisher Scientific). To visualize total proteins, the gel was stained with a Blu Coomassie solution. Protein concentration was evaluated by the Bradford reagent (Bio-Rad Laboratories, Milan, Italy). Aliquots (20 μg of proteins) from each CD sample were pulled for MS characterization of both the whole proteome and phosphoproteome. Similarly, pulled samples were prepared from controls.

### 4.3. Mass Spectrometry (MS)

To perform global proteome analysis, 20 µg of pulled cell lysate were loaded onto SDS-PAGE 12.5% in duplicate. Then, the lanes were divided into 8 bands and digested as previously described by Schevchenko et al., 2007 [44]. The MS analysis was performed using a Q-Exactive Orbitrap mass spectrometer (Thermo Fisher Scientific) equipped with a nano-ESI source and a hybrid quadrupole-orbitrap analyzer, associated with a nanoUltimate3000 UHPLC system (Thermo Fisher Scientific). Peptides were separated using a capillary EASY-Spray PepMap column (0.075 mm × 50 mm, 2 μm, Thermo Fisher Scientific). The following solutions were used as mobile phases: aqueous 0.1% formic acid (A) and CH_3_CN containing 0.1% formic acid (B). For the analysis of all samples, a linear gradient from 3% to 40% of B in 45 min and a 300 nL/min flow rate were set. MS spectra were acquired over an m/z range from 375 to 1500. MS/MS analyses were carried out in a dependent scan mode, based on the selection of the 5 most abundant ions for each spectrum. Fragmentation was achieved using N_2_ as a collision gas. The samples were analyzed as technical duplicates on the mass spectrometer.

### 4.4. Bioinformatic Analysis

MS data were processed using Proteome Discoverer software (2.4.1.15) (Thermo Scientific), using the human Uniprot database. To achieve protein identification, parameters were set as follows: Trypsin/LysC cleavage, a maximum of two missed cleavages, carbamidomethylation of cysteine as a fixed modification, methionine oxidation and tyrosine and serine phosphorylation as variable modifications, a false discovery rate (FDR) 0.05, and peptide and fragment tolerance at 10 ppm and 0.02 Da, respectively. A “label-free” approach was used for the quantitative analysis. Unique peptides were considered for each protein, and the statistical analysis of the results was performed using Student’s *t*-test.

Data on protein identification and quantitative analysis have been deposited in the public repository Jpost [45]. Bioinformatic analysis was performed to group all proteins to highlight the pathways that were differentially regulated between celiac and control cells. All proteins with a significant *p*-value change were considered, as the collective action was assessed, seeking to identify disrupted pathways rather than individual proteins. First, STRING (a database that contains predicted and known protein–protein interactions, https://string-db.org (accessed on 17 October 2025)) [46] was employed to evaluate potential interactions between proteins. Moreover, the Gene Ontology terms referring to proteins were analyzed using ShinyGO software (v0.741, South Dakota University), a powerful tool that was used to perform an efficient enrichment analysis, supplied with an efficient plot visualization due to its R-based structure [47]. Furthermore, processing was performed to allow the functional organization of proteins according to the GO terms. Additionally, for each GO term, all the genes related to each term were extracted (using ShinyGO software).

The Uniprot database was employed to perform the comparative alignment between the sequence from cDNA FLY58187 obtained from phosphoproteomic analysis (accession code B4DIT7, date 8 December 2025) and TG2 (accession code P21980). Then, based on the TG2 protein as a template, the sequence from FLJ58187 cDNA was modeled by using the Phyre2 software [48].

### 4.5. Western Blot Analysis

To validate MS analysis, we performed Western blots on cell lysates of three CD skin-derived fibroblasts and three controls. Protein samples were prepared as described; proteins were separated on a 10% SDS-PAGE and transferred to PVDF membranes. All primary antibodies (Aurogene s.r.l, Rome, Italy) were used at 1:1000 in 1% milk TBS-Tween buffer overnight, and relative secondary antibodies (Bio-Rad Laboratories) were used at room temperature for 1 h. Signals were revealed and registered by using the iBright system (Thermo Fisher Scientific) after incubation with ECL substrate (Merck, Milan, Italy) and analyzed with IBA iBright Analysis Software, version 5.4.0.

### 4.6. Statistical Analysis

Student’s *t*-test was used to calculate the statistical significance of differences between CD and control samples in Western blot experiments. The difference was regarded as statistically significant when *p* < 0.05.

## Data Availability

Proteomic data are available at the JProt repository. ID: JPST004404 (URL https://repository.jpostdb.org/preview/1191688552698e46c628f57 (accessed on 11 February 2026); access key: 2687) and ID: JPST004399 (URL https://repository.jpostdb.org/preview/1986924048698e472e66dc0 (accessed on 12 February 2026); access key: 4435). Data not presented in this manuscript are available on request from the corresponding author.

## Supplementary Materials

The following supporting information can be downloaded at: https://www.mdpi.com/article/10.3390/ijms27093938/s1.

## Abbreviations

The following abbreviations are used in this manuscript:

CD	Celiac disease
CCT8	T-complex protein 1 subunit theta
CHMP7	Charged multivesicular body protein 7
CLIC4	Pro-chloride intracellular channel protein 4
DEPs	Differently expressed proteins
ER	Endoplasmic reticulum
GOPC	Golgi-associated PDZ and coiled-coil motif-containing protein
MFN1	Mitofusin-1
MS	Mass spectrometry
MYLK	Myosin light chain kinase
NMT1	Glycylpeptide N-tetradecanoyltransferase 1
SNCG	γ-synuclein
TAGLN3	Transgelin-3
TG2	Type 2 transglutaminase
