# Supplementary material for: Proteomic Analysis Highlights Peculiar Protein and Phosphoprotein Profiles in Dermal Fibroblasts from Celiac Disease Patients
Source: Int J Mol Sci. 2026 Apr 28;27(9):3938. doi: 10.3390/ijms27093938 (PMC13163242; doi:10.3390/ijms27093938)
Supplement: Supplementary file 1 [file ijms-27-03938-s001.zip › Supplementary Figures.pdf]

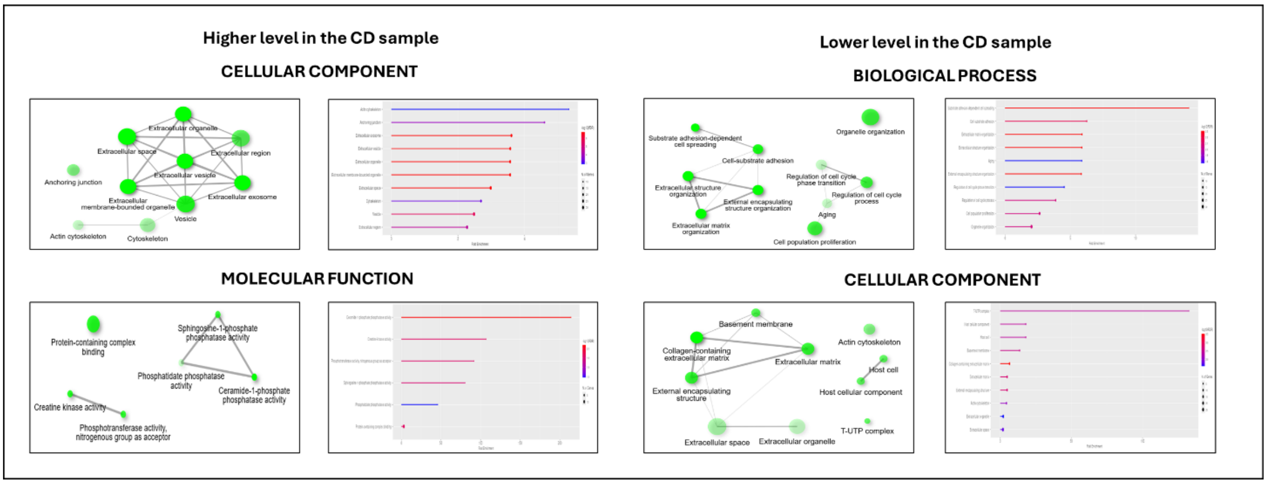

Supplementary Figure 1

Results of Gene Ontology analysis of over-abundant or under-abundant proteins in CD samples. ***(Left panel)*** Proteins whose abundance was significantly higher in CD cells than in control cells were grouped based on the cellular component in which they are primarily localized and their molecular functions. The Gene Ontology analysis did not reveal any predominant biological process in which they are involved. (**Right panel**) Proteins whose abundance was significantly lower in CD cells compared to control cells were grouped based on the biological processes in which they are involved and the cellular component in which they are primarily localized. The Gene Ontology analysis did not reveal any prevalent molecular function shared by these proteins.

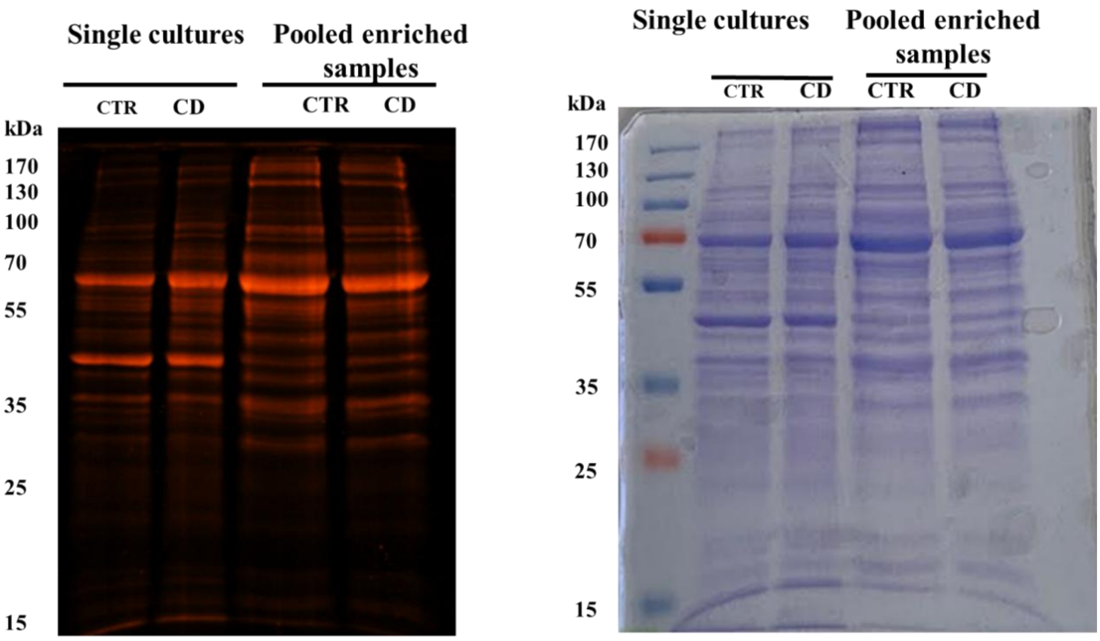

Supplementary Figure 2

Protein profiles of phosphoproteins after the enrichment step. After affinity chromatography on total protein samples from four pooled control (CTR) and four pooled CD fibroblast cultures, proteins from single representative cultures and

from enriched pooled samples (40 µg) were separated onto a denaturant 10% polyacrylamide gel. Gel was stained with Pro-Q® Diamond for phosphoprotein (left panel) and Coomassie Blue for total protein (right panel).

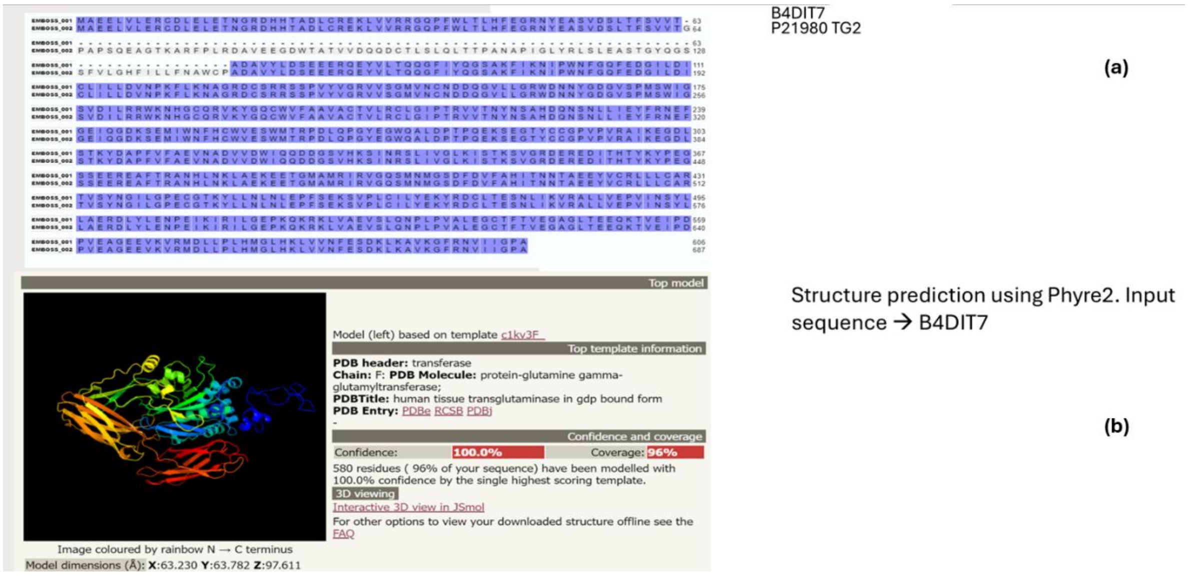

Supplementary Figure 3

Alignment and structural prediction of the flj58187 transcript identified by mass spectrometry analysis.
(**a**) Sequence alignment between the amino acid sequence derived from the flj58187 cDNA and the sequence of type 2 tranglutaminase (TG2) protein (Uniprot code P21980). (**b**) Structural prediction of the amino acid sequence using Phyre2 software.
